# Identifying key genes of classic papillary thyroid cancer in women aged more than 55 years old using bioinformatics analysis

**DOI:** 10.3389/fendo.2022.948285

**Published:** 2022-09-02

**Authors:** Chang-Chun Li, Muhammad Hasnain Ehsan Ullah, Xiao Lin, Su-Kang Shan, Bei Guo, Ming-Hui Zheng, Yi Wang, Fuxingzi Li, Ling-Qing Yuan

**Affiliations:** ^1^ National Clinical Research Center for Metabolic Diseases, Department of Metabolism and Endocrinology, The Second Xiangya Hospital, Central South University, Changsha, China; ^2^ Department of Radiology, The Second Xiangya Hospital, Central South University, Changsha, China

**Keywords:** thyroid carcinoma, R package, gene ontology, Kyoto Encyclopedia of Genes and Genomes, protein–protein interaction, biomarkers, prognosis thyroid carcinoma, prognosis

## Abstract

**Background:**

The incidence rate of thyroid carcinoma (THCA) markedly increased in the recent few decades and has been likely over-diagnosed, especially papillary thyroid cancer (PTC) in women. However, the incidence of advanced-stage papillary thyroid cancer is also rising. According to earlier studies, tumors with identical pathology might have different clinical outcomes, which implies some variances in papillary thyroid cancer. Although the mortality of thyroid cancer has remained stable or declined, there is still an important problem in estimating whether it is benign or needs surgery for patients with papillary thyroid cancer.

**Methods:**

After obtaining data from The Cancer Genome Atlas (TCGA) Project-THCA database by R package TCGA bio links, 18 samples (11 at stage IV as high-risk group and 7 at stage I as low-risk group) were obtained using survival package and edgeR to ensure differential expression; ClusterProfiler package was used to carry on gene set enrichment analysis and searched the possible pathways in the Kyoto Encyclopedia of Genes and Genomes (KEGG) database. STRING and Cytoscape were used to construct and modify the protein–protein interaction (PPI) network to get hub genes of differentially expressed genes. Next, the pROC package was used to get the receiver operating characteristic (ROC) curves of hub genes’ disease-free survival (DFS). Then, transcription factors (TFs) and miRNAs of key genes were predicted by ENCORI and AnimalTFDB. In the end, TF–target genes–miRNA regulatory network was also constructed by Cytoscape.

**Results:**

Our research obtained the top 9 candidate genes from the whole network (IFNA1, MRC1, LGALS3, LOX, POSTN, TIMP1, CD276, SDC4, and TLR2). According to the ROC results, TIMP1, LOX, CD276, IFNA1, TLR2, and POSTN were considered to play a more critical role in malignant papillary thyroid cancer or immature cancer of papillary thyroid cancer. Our analysis concludes that TIMP1, LOX, CD276, IFNA1, TLR2, and POSTN are identified as thyroid cancer biomarkers, which lead to the different clinical courses of a woman older than 55 years old with papillary thyroid cancer. Especially CD276, POSTN, and IFNA1 may be considered as new biomarkers associated with the prognosis of thyroid cancer.

**Conclusions:**

TIMP1, LOX, CD276, IFNA1, TLR2, and POSTN have different expressions in PTCs, which lead to the various clinical courses of a woman older than 55 years old with papillary thyroid cancer. Especially CD276, POSTN, and IFNA1 may be considered as new potential biomarkers associated with the prognosis of thyroid cancer. In addition, TF–miRNA–target gene regulatory network may help further reach for PTC.

## Introduction

The incidence rate of thyroid carcinoma (THCA) markedly increased in the recent few decades (1) and has been likely over-diagnosed because ultrasonography or other modern diagnostic techniques have been widely used to discover previously undetectable thyroid nodules (2). Differentiated thyroid cancer is the most common thyroid cancer, particularly the category of papillary thyroid cancer (PTC) in women diagnosed by methods such as fine-needle aspiration (FNA) after imaging tests. However, the incidence of advanced-stage PTC (large tumors and locally advanced and/or metastatic) rises (3). The fact that tumors with identical pathologies might have distinct clinical outcomes suggests some variances in PTC, according to previous studies (4, 5). Although the mortality of THCA has remained stable or declined, there is still an important problem in estimating if it is benign or needs surgery for patients with PTC.

Controversial issues in thyroid cancer management mainly focus on treatment options for different thyroid cancers (DTCs) (6). Recently, detecting genetic changes in thyroid cancer has been generally stressed and applied as an essential means to guide treatment (7). DNA sequencing technology has been frequently used to detect a different group of diagnoses. Bioinformatics technology has also been employed to identify those different gene expressions (DEGs). The BRAF and RAS family’s mutations are usually found in THCA and mitogen-activated protein kinase (MAPK) cellular signaling pathway and PI3K/AKT/mTOR pathway (8, 9). Those findings have greatly improved our understanding of studying THCA with different clinical outcomes.

This research performed many analyses through a wide-ranging bioinformatics study to classify the critical hub gene in thyroid cancer with a poor prognosis. A total of 994 genes upregulated in the high-risk group were obtained from TCGA-THCA data by edgeR (10, 11). Then, GO and KEGG pathway enrichment and the construction of protein–protein interaction (PPI) network and survival analysis were performed in these differentially expressed genes (DEGs) (12–14). Finally, key genes, transcription factors, and miRNA relations that might be important in THCA with poor prognosis were identified.

## Methods

### Datasets selection and DEGs identification

The Cancer Genome Atlas (TCGA) research network has collected many public clinical and molecular profiling of more than 10,000 tumor patients across 33 different tumor types. We obtained the clinical and transcriptome profiling of THCA from the TCGA-THCA database by TCGA-biolinks packages, which provide several useful functions to search, download, and prepare TCGA samples for data analysis (15), using the following criteria: (A) PTC-classic/usual, (B) female, (C) age ≥55 years old, (D) datasets including stage I and IV samples, and (E) replicated samples value being saved with unique bcr patient barcode. Next, Cox proportional hazards model was performed by survival packages of R for those data, and the result was presented by forest plot packages (16, 17). Furthermore, the edgeR packages were used to distinguish the DEGs in each sample in R (Version 4.1.2) (10, 11). The standard for DEGs has been considered a false discovery rate of <0.05. The groups’ criteria were |log2FC (fold change) | ≥ 1.

### GO and KEGG enrichment analyses of DEGs

Gene Ontology (GO) and Kyoto Encyclopedia of Genes and Genomes (KEGG) were performed to reveal the functional enrichment analysis of DEGs. BiomaRt packages that include over 800 different biological datasets spanning were used to convert gene ID from other types (18, 19). ClusterProfiler packages that automate enrichment analysis of gene clusters were used to progress GO enrichment analyses, which include cellular component (CC), biological process (BP), and molecular function (MF), and KEGG pathways analysis (20, 21). Finally, GOplot and ggplot2 packages were used to make figures of those results (22, 23).

### PPI network construction

STRING (https://string-db.org/), an online database, was used to establish the protein–protein interaction (PPI) network of the candidate genes, which evaluates the interacting units with protein-coding gene loci (represented by their main, canonical protein isoform) (24), and the minimum required score was set as the medium confidence (0.400). Moreover, Cytoscape (Version 3.9.1) was used to visualize, model, and analyze molecular and genetic interaction networks (25). Next, the analysis network of Cytoscape tool, Molecular Complex Detection (MCODE), was applied to screen modules of the PPI network with a degree cutoff = 2, node score cutoff = 0.2, k-core = 2, and max. depth = 100.

### Survival analysis

The receiver operating characteristic (ROC) curve is a commonly used graphical summary to evaluate the predictive value of biomarkers (26). To verify the prognosis results of hub genes, disease-free survival (DFS) data obtained from TCGA and pROC package were used to make the ROC curve figure (27).

### TF and miRNA regulatory network construction

After the reference sequence (RefSeq) of the key genes were obtained from National Center for Biotechnology Information (NCBI) RefSeq database (https://www.ncbi.nlm.nih.gov/), transcription factors were predicted by AnimalTFDB (https://bioinfo.life.hust.edu.cn/AnimalTFDB/#!/) and selected by score >20 (28, 29). ENCORI (https://rna.sysu.edu.cn/encori/index.php) was used to predict miRNAs that bind to hub genes and apply standards including CLIP-Data ≥3, pan-cancer ≥1, and miRNA, with the least intersections in three databases selected (30).

## Results

### Patient characteristics

**Figure 1:**
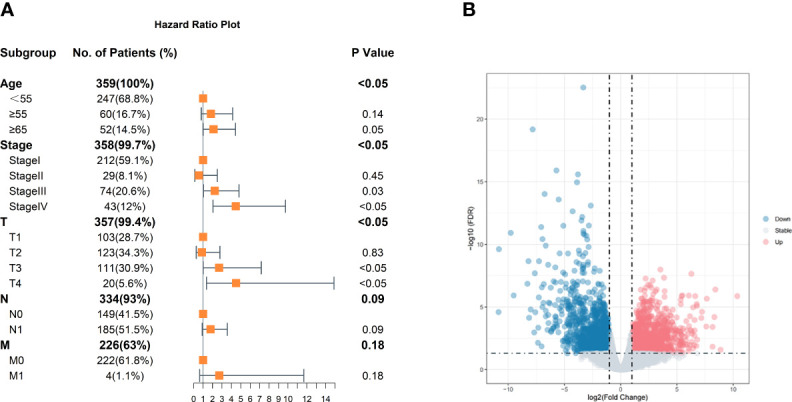
Clinical characteristics of classic PTC and volcano plot of DEGs. **(A)** Age, stage, and tumor size are significant. The reference of age is <55 years old. The reference of stage is stage I, and references of TNM are T1, N0, and M0. **(B)** Volcano plot of DEGs. Those blue dots represent downregulation in high-risk group thyroid carcinoma compared with low-risk group samples; red represents upregulation, and gray dots show no significant difference in the two groups.

The clinical characteristics of 359 cases of classic PTC were obtained from the TCGA-THCA project, including days to the last follow-up, days to death, disease-free status, age at diagnosis, TNM classification, and tumor stage. After Cox analysis, age at diagnosis and tumor stage were significant for disease-free status (**Figure 1A**). Compared to age <55 years, the order indicated a poor prognosis that corresponds with previous research. At the same time, ages between 55 and 65 still have no noticeable difference with age <55 years old, suggesting that there might some of the patients with a preferable prognosis. Consequently, with age more than 55 years, 7 cases of stage I as a low-risk group and 11 cases of stage IV as a high-risk group were finally obtained after filtering the following criteria. The different expressions between the two groups were analyzed by edgeR and shown in the volcano plot (**Figure 1B**).

### Function annotation of DEGs

**Figure 2:**
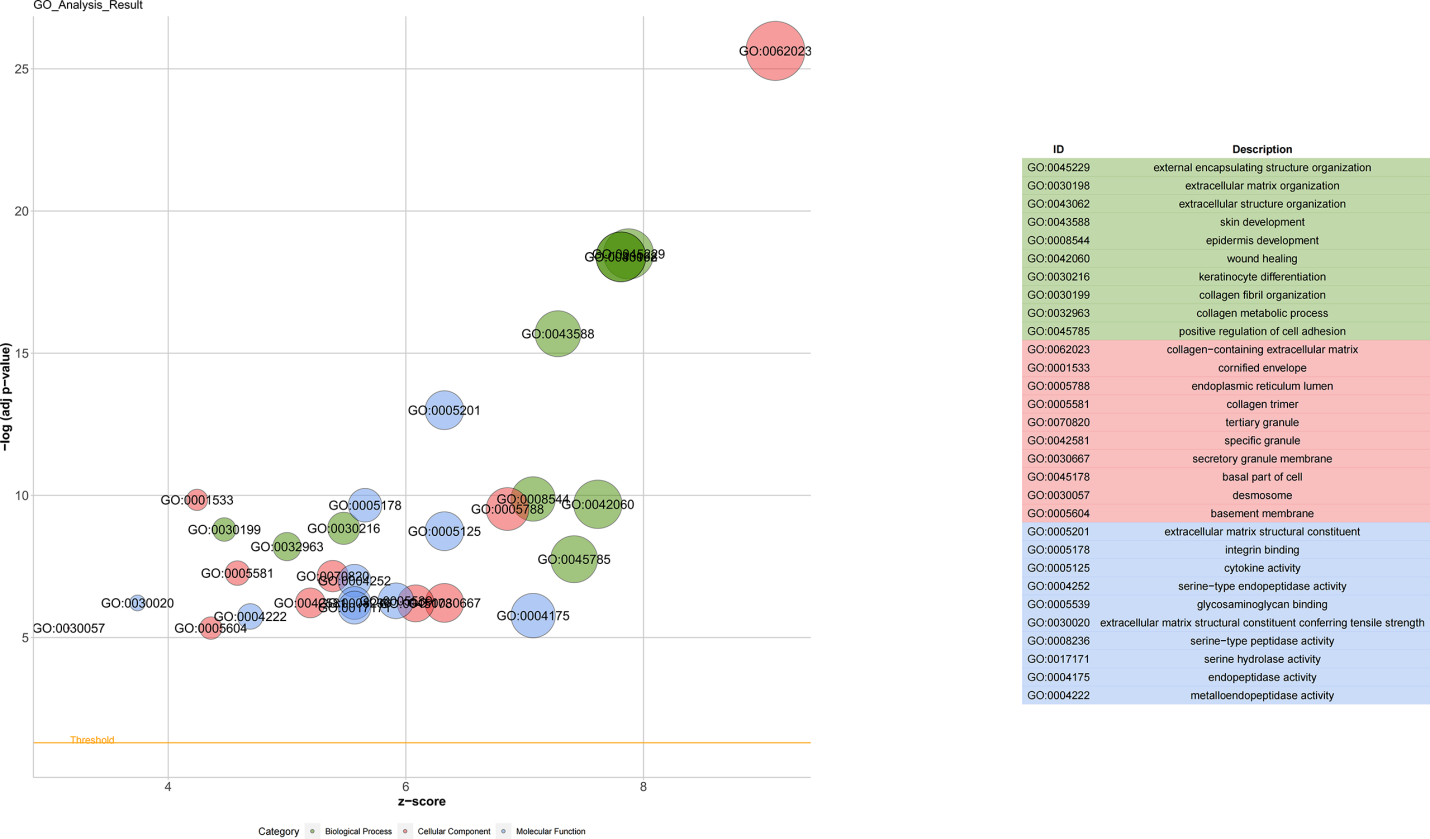
Gene Ontology analyses of differentially expressed genes. Biological process (BP) was enriched in the external encapsulating structure organization, extracellular matrix organization, and extracellular structure organization. The cellular component (CC) was enriched in the collagen-containing extracellular matrix, cornified envelope, and endoplasmic reticulum lumen. Molecular function (MF) was enriched in extracellular matrix structural constituent, integrin binding, and cytokine activity.

GO enrichment analysis was completed to explore the biological functions of all DEGs of the upregulated gene obtained after edgeR. The GO term biological process analysis corroborated that the DEGs were enriched in external encapsulating structure organization, extracellular matrix organization, and extracellular structure organization. The GO term cellular component was enriched in the collagen-containing extracellular matrix, cornified envelope, and endoplasmic reticulum lumen. Apart from that, the molecular function classification of GO was enriched in extracellular matrix structural constituent, integrin binding, and cytokine activity (**Figure 2**).

**Figure 3:**
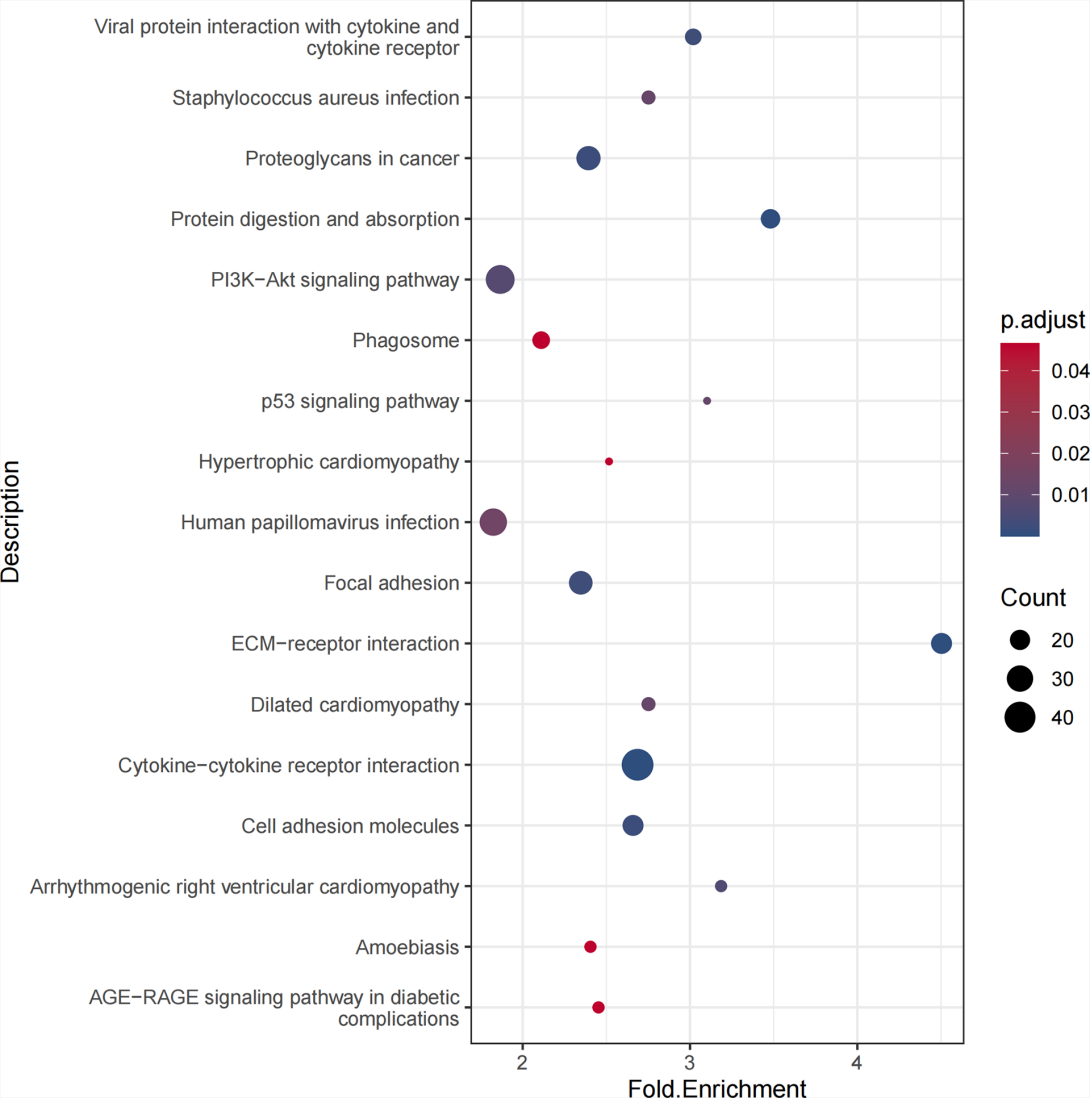
KEGG pathways of differentially expressed genes. The KEGG pathway could find proteoglycans in cancer, PI3K-Akt signaling pathway, and p53 signaling pathway related to thyroid carcinoma.

The enrichment analysis of the KEGG pathway demonstrated that DEGs were correlated with proteoglycans in cancer, PI3K-Akt signaling pathway, and p53 signaling pathway (**Figure 3**).

### Construction of PPI network

**Figure 4:**
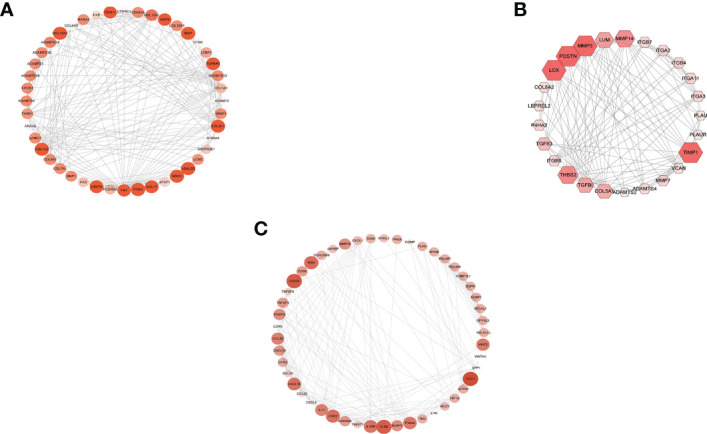
MCODE cluster. Cluster calculated by MCODE after protein–protein interaction network constructed, and different sizes and colors represent different MCODE scores. **(A)** Cluster 1 (score, 10.1). **(B)** Cluster 2 (score, 9.8). **(C)** Cluster 3 (score, 8.5).

The STRING database identified the interaction of these upregulated DEGs. The combined score >0.4 (medium confidence score) was considered statistically significant. Then, a PPI network was made with Cytoscape. A total of 905 nodes and 4,941 edges were included in that PPI network. Additionally, the modules in the PPI network were analyzed by MCODE, and >8 was set as cutoff criteria with the default parameters (degree cutoff, 2; node score cutoff, 2; K-score, 2; and max depth = 100) (**Figure 4**). Next, the top 3 genes of every cluster (IFNA1, MRC1, LGALS3, LOX, POSTN, TIMP1, CD276, SDC4, and TLR2) were obtained as hub genes after being ranked by MCODE score.

### Survival analysis

**Figure 5:**
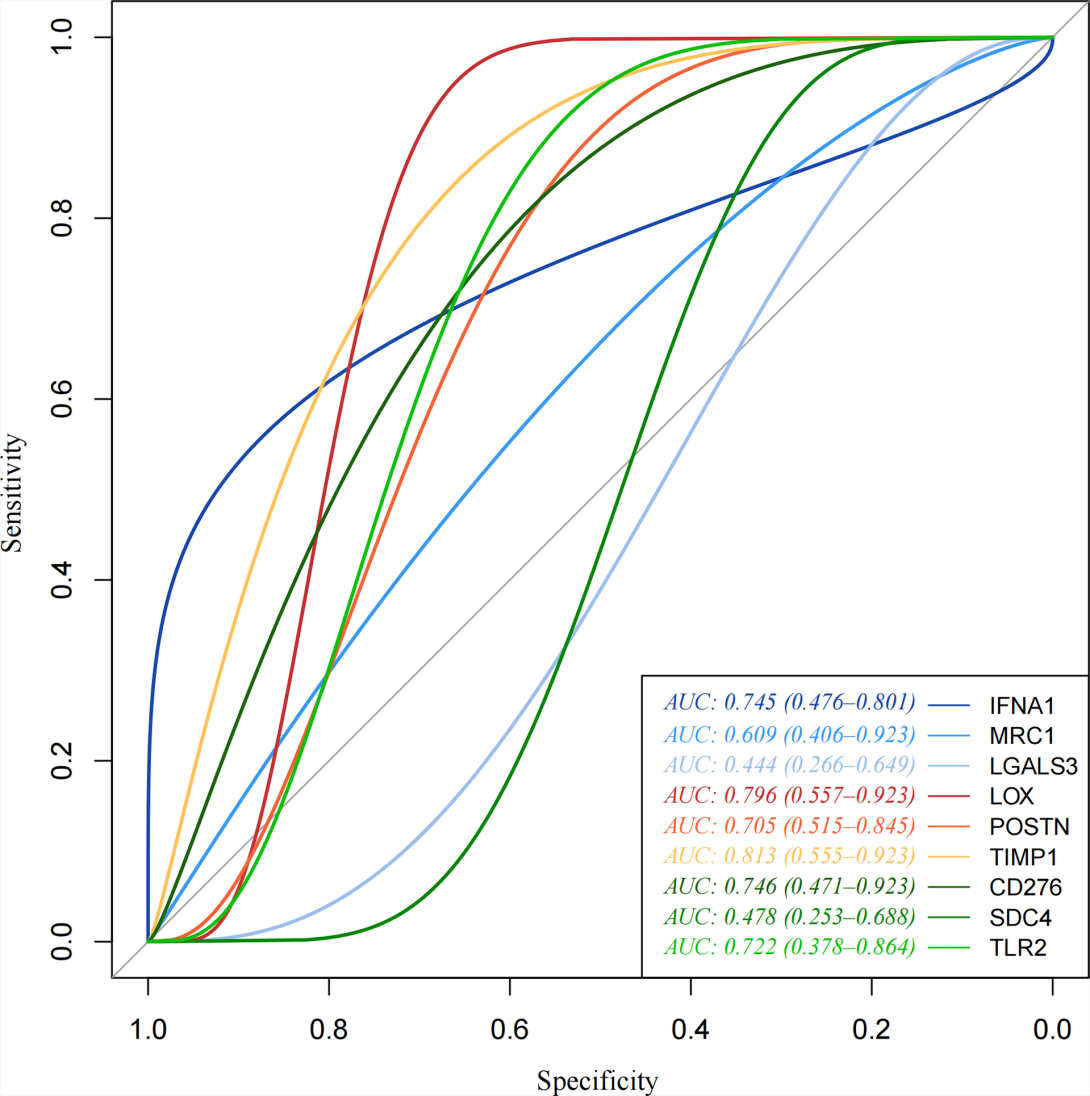
Receiver operating characteristic (ROC) curve. Receiver operating characteristic (ROC) curve made by pROC. TIMP1, LOX, CD276, IFNA1, TLR2, and POSTN as key genes are more relevant to THCA.

**Figure 6:**
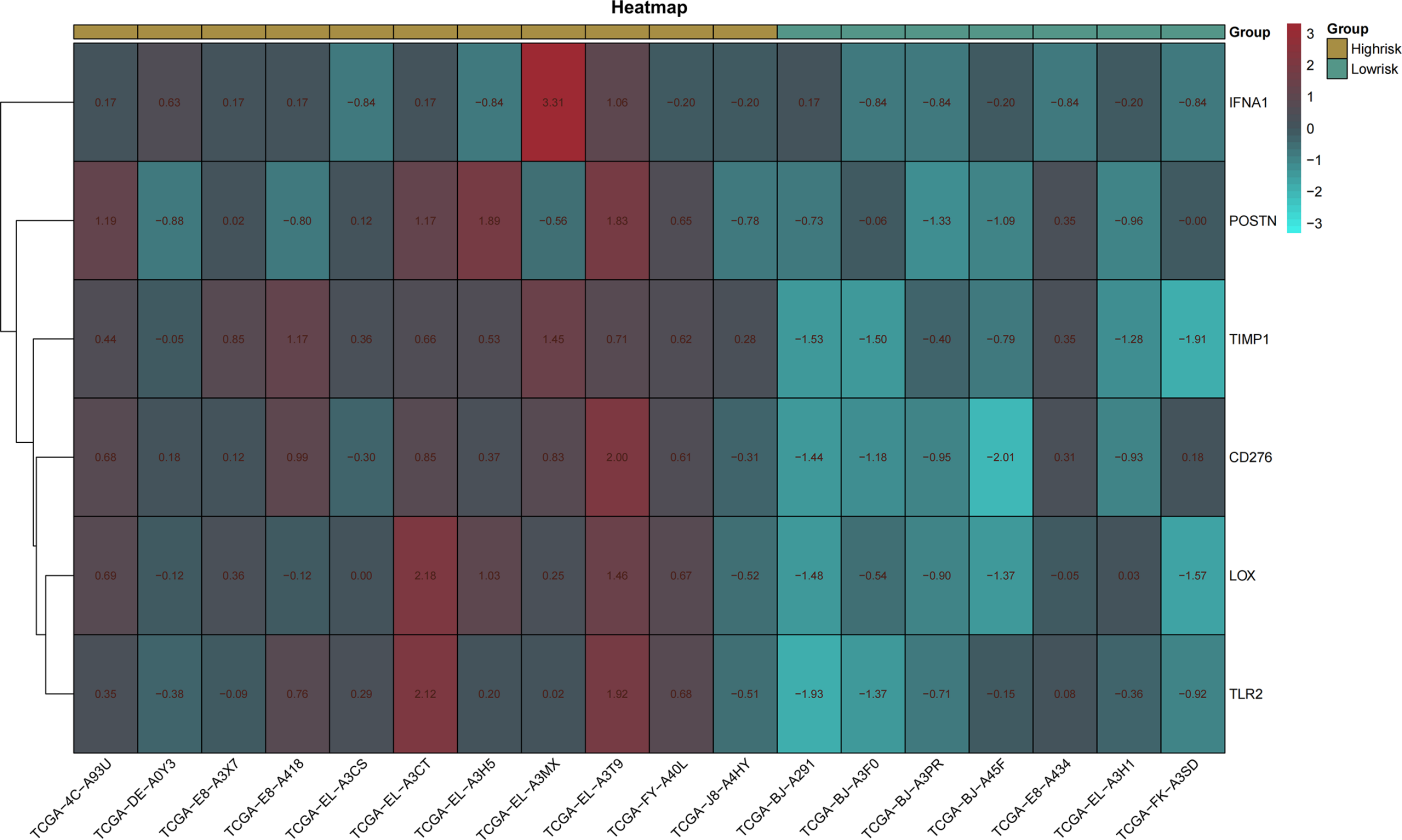
Heatmap of key genes. Heatmap made by pheatmap package and normalized by scale function. Different expression of genes in the high- and low-risk groups.

pROC packages made the receiver operating characteristic (ROC) curve to explore the patient’s prognosis with high-expression hub genes. According to the results of the ROC curve, TIMP1, LOX, CD276, IFNA1, TLR2, and POSTN as key genes are more relevant to THCA (**Figure 5**). The heatmap of key gene expression in sample patients was made after normalization. Compared to IFNA1, high expressions of other genes are more common in the high-risk group (**Figure 6**).

### TF and miRNA regulatory network

**Figure 7:**
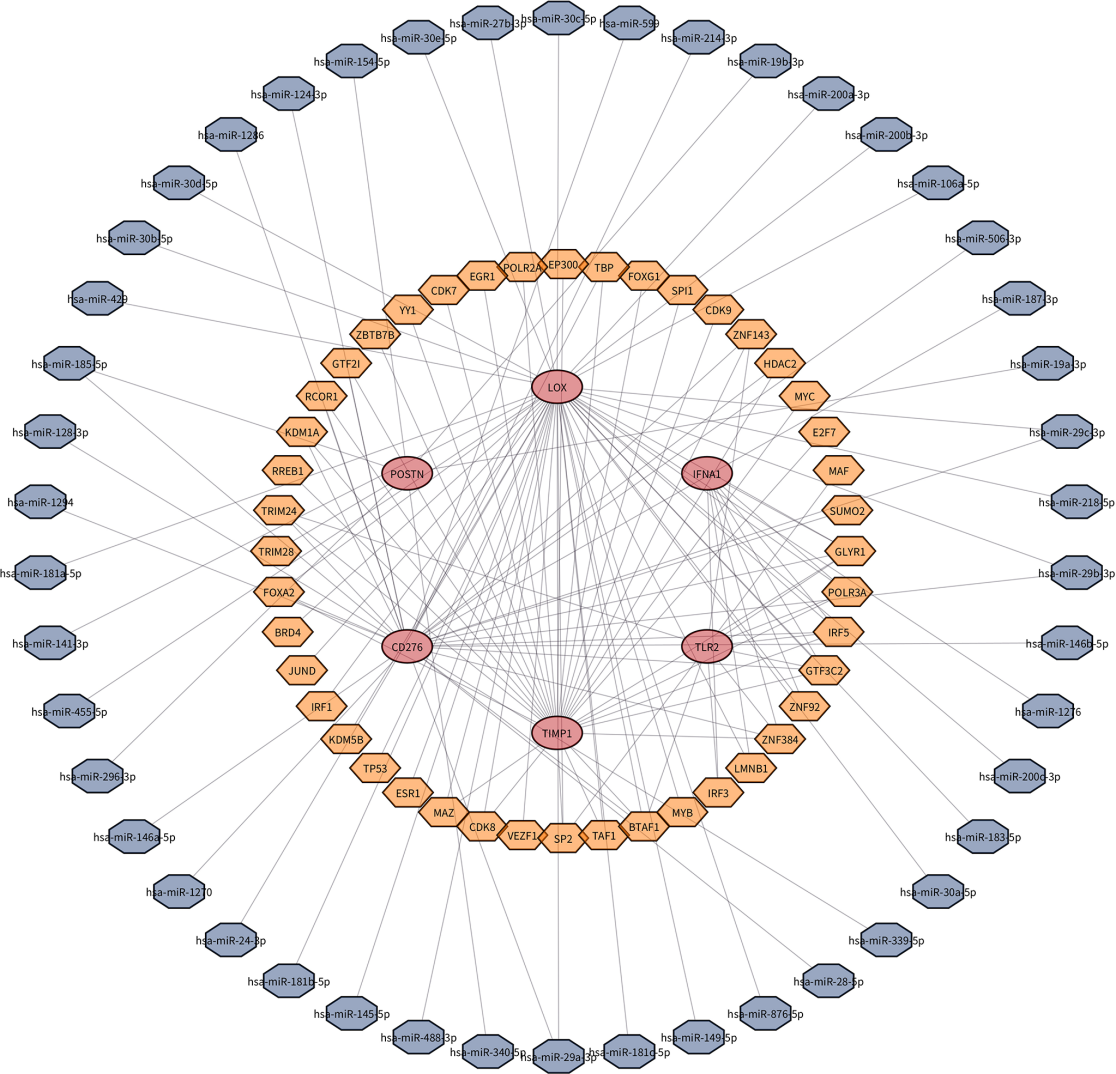
TF–miRNA–target gene network. A total of 47 miRNAs and 44 transcription factors that could be bound to key genes are predicted.

A total of 47 miRNAs and 44 transcription factors that could be bound to key genes are predicted. TF, miRNA, and target gene relations were revealed in the regulatory network (**Figure 7**).

## Discussion

In recent years, thyroid carcinoma has had a worldwide and dramatic incidence, especially in women. However, thyroid cancer treatment options are limited, and they mostly need to have surgery and supply of thyroxine for the whole of their later life. The high-intensity treatment for most tumor diameters over 1 cm, especially patients with differentiated thyroid cancer or papillary thyroid cancer, has also been controversial after PTC prognosis is more remarkable than that of other tumors. The fact that advanced thyroid cancer is found with the increase in cardinality estimate also promotes more research to distinguish the difference from PTC so that more suitable and precise therapeutics could be suggested to each of the patients.

For the above target, it is critical to know the prognosis of carcinoma, especially for PTC, which has a high risk of incidence and is likely over-diagnosed. Many studies have utilized bioinformatics technology to uncover biomarkers in THCA or PTC. However, the majority of them compared carcinoma and normal samples. According to the 8th American Joint Committee on Cancer (AJCC) and other experts, there may be distinct influencing factors for varying clinical outcomes in patients with PTC who are over 55 years old (31–34). In the current study, based on the hypothesis of difference in PTC, we explore the potential difference between PTC classification in women over 55 years old who have the worst prognosis and those with favorable forecasts based on data from TCGA.

In the present study, 994 upregulated genes were obtained from TCGA-THCA data using the edgeR package. GO was enriched in cell connection and extracellular matrix organization. KEGG pathway enrichment analysis of those DEGs could be linked to thyroid cancer growth and metastasis pathways such as proteoglycans in cancer, PI3K-Akt signaling pathway, and p53 signaling pathway. After using MCODE and Cytoscape network analysis tools of Cytoscape, nine hub genes with greater degrees were finally obtained (IFNA1, MRC1, LGALS3, LOX, POSTN, TIMP1, CD276, SDC4, and TLR2), which were considerably overexpressed in PTC with a poor prognosis compared to those with a better prognosis. In comparison to the survival analysis of nine hub genes, TIMP1, LOX, CD276, IFNA1, TLR2, and POSTN as key genes are more relevant to THCA.

Tissue inhibitor of metalloproteinase 1 (TIMP1) belongs to the TIMP gene family, and its encoded protein can promote cell proliferation. TIMP1 was found to be overexpressed in classic and follicular variants of PTC in different age and gender groups and in the BRAF-MUT group compared to that in BRAF-WT patients in previous research (35, 36). It binds CD63 on the cell surface membrane and activates AKT signaling pathway to make antiapoptotic activity and predict aggressive behaviors (37, 38). Lysyl oxidase (LOX) was found to be overexpressed in aggressive cancers and related to MMPs and TIMPs by regulating SNA12 expression (39). In addition, LOX upregulation is also associated with anaplastic thyroid cancer progression and aggressive tall cell variant of PTC (TC-PTC) compared with the differentiated thyroid cancers [classic PTC (cPTC) and follicular variant of PTC (FV-PTC)] that may respond to BRAF activation (40–42). Toll-like receptor 2 (TLR2) is a member of the Toll-like receptor (TLR) family; it was found to be mediated by Akt phosphorylation by GT1b (trisialoganglioside 1b) and activated PI3K/Akt signaling pathway (43). POSTN-encoded protein binds to integrins to support adhesion, migration of epithelial cells, and participation in cancer stem cell maintenance and metastasis. Previous research once found a wide variability of POSTN expression in a different histological subtype of PTC, and high stromal POSTN expression is associated with aggressive tumor behavior (44). Compared to TIMP1 and LOX, the relation between TLR2 and thyroid carcinoma was unclear, especially in PTC. However, previous research indicated that overexpression of TLR2 was associated with a high risk of colorectal cancer, cervical cancer, and oral cancer (45–48). CD276, also known as B7-H3, has upregulated expression in many cancer cells and related with tumor cells by pathological angiogenesis (49). CD276 has been found to have an upregulated expression at the protein and mRNA levels in different types of thyroid cancer, and its effect on thyroid carcinoma has also been researched recently. CD276 was overexpressed in advanced thyroid carcinoma such as ATC, which is consistent with our results; its high expression indicates a poor prognosis of different PTCs (50, 51). We found that in the classic PTC, the upregulated CD276 is also related to a poor prognosis. Periostin (POSTN) encoded a secreted extracellular matrix protein and is found to exhibit stromal deposition in invasive portions and cytoplasmic expression of undifferentiated thyroid carcinoma cells (52). Periostin has different expressions in different kinds of PTCs, and upregulated periostin could also be found in PTC compared to non-neoplastic tissues. Upregulation of periostin-introduced invasion and lymph node metastasis may be due to loss of cellular polarity/cohesiveness and epithelial–mesenchymal transition (EMT) (53). This research also was consistent with the above results and found that in classic PTC of female patients aged more than 55 years, POSTN still has a different expression and upregulated POSTN still indicated a poor prognosis. Interferon alpha 1 (IFNA1) could encode a member of the type I interferon, and expression of IFNA1 is associated with HPV-positive head and neck cancers (54). Interferon alpha 1 could be found in normal thyroid cells and differentiated thyroid cancer and also used in some diseases, but long-term interferon (IFN) therapy is frequently associated with side effects on the thyroid gland (55). Although its effect is considered antiproliferative, interferon alpha is also found to influence malignant thyroid cancer by the microenvironment or affect other factors, such as tumor necrosis factor (TNF) or epidermal growth factor (EGF) (56, 57). According to our research, IFNA affects high-risk group PTC with an upregulated expression. More facts about its effect on PTC also need to be explored by further research. Above all, in our results, TIMP1, LOX, and POSTEN had different expressions no matter what type of thyroid cancer and papillary thyroid cancer; even in pure classic PTC, high expression of those genes also predicted a poor prognosis. Although CD276, TLR2, and IFNA1 were not found to have complex functions in thyroid cancer, they all participate in different carcinomas. These results indicate that further study is needed to reveal their roles in thyroid carcinoma.

## Data availability statement

The original contributions presented in the study are included in the article/supplementary material. Further inquiries can be directed to the corresponding author.

## Author contributions

L-QY designed the analyses. C-CL and MU analyzed the data and wrote the manuscript. XL and S-KS collected the data, YW and FL prepared the figures, and BG and M-HZ revised the manuscript. All authors contributed to the article and approved the submitted version.

## Funding

This work was supported by grants from the National Natural Science Foundation of China (Nos. 82100944, 82100494, 82070910, and 81870623), Natural Science Foundation of Hunan Province (No. 2021JJ40842), and Key R&D Plan Hunan Province (2020SK2078).

## Acknowledgments

We want to thank our colleagues in the lab for their assistance.

## Conflict of interest

The authors declare that the research was conducted in the absence of any commercial or financial relationships that could be construed as a potential conflict of interest.

## Publisher’s note

All claims expressed in this article are solely those of the authors and do not necessarily represent those of their affiliated organizations, or those of the publisher, the editors and the reviewers. Any product that may be evaluated in this article, or claim that may be made by its manufacturer, is not guaranteed or endorsed by the publisher.
